# Multi-omic characterisation as a tool to improve knowledge, valorisation and conservation of wild fruit genetic resources: the case of *Arbutus unedo* L

**DOI:** 10.3389/fpls.2023.1195673

**Published:** 2023-09-08

**Authors:** Maria Tartaglia, Pierpaolo Scarano, Antonello Prigioniero, Daniela Zuzolo, Alessia Postiglione, Alessandra Falzarano, Angela Amoresano, Anna Illiano, Gabriella Pinto, Rosario Schicchi, Anna Geraci, Rosaria Sciarrillo, Carmine Guarino

**Affiliations:** ^1^Department of Science and Technology, University of Sannio, Benevento, Italy; ^2^Department of Chemical Science, University of Naples Federico II, Naples, Italy; ^3^INBB - Consorzio Interuniversitario Istituto Nazionale di Biostrutture e Biosistemi, Rome, Italy; ^4^Department of Agricultural, Food and Forest Sciences (SAAF), Università degli Studi di Palermo, Palermo, Italy; ^5^Department of Biological, Chemical and Pharmaceutical Sciences and Technologies (STEBICEF), University of Palermo, Palermo, Italy

**Keywords:** strawberry-tree, *Arbutus unedo* L., proteomics, pedoclimatic metabolomic imprinting, ripening

## Abstract

The valorisation and conservation of plant genetic resources (PGRs) and wild fruit PGRs are critical to ensure the maintenance of genetic and cultural heritage and to promote new perspectives on resource use. New strategies to characterize PGRs are needed, and the omics approach can provide information that is still largely unknown. The Strawberry tree (*Arbutus unedo* L.) is an underutilized, drought and fire-resistant species distributed in the Mediterranean area and its berries have large ethnobotanical use. Although their phenolic profile and antioxidant capacity are known, they are not well characterised, particularly from a proteomic perspective. The aim of this work is the characterisation of two ecotypes of *A. unedo* (Campania and Sicily) from a molecular viewpoint to valorise and encourage the preservation of this wild fruit. Samples were collected from two different geographical areas to assess whether different geographical conditions could influence the characteristics of leaves and fruits at the three stages of ripening (green, veraison, red). Proteomic analysis identified 904 proteins, of which 122 showed significance along the ripening. Some of these differentially abundant proteins, such as chalcone synthase, show a marked increase during ripening. The protein functional classes with the highest representation are involved in protein and amino acid metabolism, glycolysis and in secondary metabolism. From a proteomic perspective, there are no differences between the fruits from the two regions compared by the ripening stage. However, the pedoclimatic metabolic imprinting allowed the observation of good diversity in the metabolomic profiles between the two ecotypes, especially for anthocyanins, 4 times more abundant in the Sicilian veraisoned fruit than in the Campania one, and catechins, with double the abundance in the Campania ecotype compared to the Sicilian ecotype in the green phase, but more abundant (3x) in the Sicilian veraisoned fruit. Phenolic compounds show a 20% greater abundance in the Campania green arbutus fruit than in the Sicilian one, values that then equalise as ripening progresses. Multi-omic characterisation enhanced the knowledge on a wild fruit plant species which shows specific adaptations and responses to the environment to be considered when addressing the issue of local agrobiodiversity.

## 1 Introduction

Plant genetic resources (PGRs) represent living material containing genetic information of current and potential value to humankind, becoming an asset for its current and future survival (Dulloo, 2018; Singh et al., 2020). To date, a large part of the world’s population bases its survival on a dozen plant species widely cultivated and standardised worldwide (Jones et al., 2021), neglecting fruit and horticultural wild species related PGRs. Wild plants are often underutilised, and the lack of interest in them puts these PGRs at serious risk, despite it is potentially possible to search their gene pool for the key to increasing the resilience of our agri-food system (Rymbai et al., 2016; Dulloo, 2018; Singh et al., 2020). The characterisation of wild plant species at risk of cultural erosion is an extension of the concept of PGRs conservation. It refers especially to plant species, not strictly of agricultural interest, but deeply linked to local cultural and traditional heritage. These represent genetic resources adapted to the environment and a source of bioactive molecules not yet fully known and exploited (Giupponi et al., 2021). To preserve rural biodiversity, ecosystem services, and environmental quality, it is essential to understand and value the intrinsic relationship between the rural landscape and biodiversity conservation. Anthropogenic activities have negatively impacted rural biodiversity, and traditional land management approaches have been abandoned in favour of more economically rewarding monocultural production models (Biasi et al., 2015). *Arbutus unedo* L. *(Strawberry tree)* is a fruit tree species, belonging to the Ericaceae family, which distribution area is Mediterranean. It provides edible fruits which are usually used to produce alcoholic beverages and distillates, sweets, jams and jellies, instead of being eaten fresh (Pallauf et al., 2008; Oliveira et al., 2011a). Strawberry tree leaves and fruits have been widely used in traditional medicine, since the Greek civilisation, thanks to the well-known antimicrobial, antioxidant, diuretic, antiseptic and laxative effects (Pabuçcuoǧlu et al., 2003; Oliveira et al., 2009; Malheiro et al., 2012). The cultural value associated with the consumption of semi-wild and wild edible plants is often associated with their contribution to the local communities’ health that have inherited their use (Oliveira et al., 2011b; Morales, 2022). However, the cultivation of strawberry trees has been gradually replaced by species with greater economic value, restraining its use as an ornamental plant. The content of phenolic compounds and the antioxidant potential of strawberry tree leaf extracts have been evaluated in several studies (Maleš et al., 2006; Oliveira et al., 2009; Malheiro et al., 2012), whereas the potential bioactive components of strawberry tree fruit have been less investigated (Pallauf et al., 2008; Ruiz-Rodríguez et al., 2011; Ruiz-Rodríguez et al., 2014), above all in consideration of the physiological alterations due to the ripening process which alters the fruit biochemical characteristics (Oliveira et al., 2011a). Furthermore, no proteomics data for *A. unedo* fruit was currently reported in the literature. The valorisation of a species, whose cultural value exceeds its economic potential, passes through its characterisation This must be considered in relation to the pedogeographical conditions of the niche in which the species lives under natural conditions.

The aim of the work are (i) the proteomic and metabolomic characterisation of the *A. unedo* fruit during the ripening phases (G = fully developed but still green fruit, V = ripening fruit with a colour change from yellow to red/orange, R = completely ripe fruit), (ii) the metabolomic characterisation of *A. unedo* leaves, (iii) the observation of potential differences found in the proteomic and metabolomic analysis on fruits and leaves sampled in two different geographical areas to evaluate the pedogeographic influence on the plant samples characteristics. Given the peculiarity of *A. unedo*, which presents in the same time leaves and fruits in the various stages of ripening between October and November, the sampling allowed the simultaneous collection of leaves and differentially ripened fruits, from two areas (Campania and Sicily).

## 2 Materials and method

### 2.1 Chemicals and reagents

Microfiltered and ultrapure water was used for the preparation of the solutions *via* the Merck Millipore ZRQS0P3FR Direct system. All solvents and reagents used in the experiments were of a high degree of purity. Ethanol ≥99.9% ACS for analysis, methanol for HPLC, anhydrous 95% n-hexane, chloroform for chromatography, 1-butanol ACS reagent ≥99.5%, hydrochloric acid 37% RPE for analysis, potassium bicarbonate, anhydrous sodium carbonate for analysis, gallic acid ACS for analysis and Sudan IV reagent were purchased from Sigma-Aldrich Chemical Company (Milan, Italy). Folin-Ciocălteu reagent and hypergrade acetonitrile for LC-MS were purchased from Merck Millipore GmbH (Milan, Italy). 2,2-diphenyl-1-picrylhydrazyl (DPPH·) was obtained from Alfa Aesar (from Thermo Fisher Scientific companies in Rodano, Milan, Italy). Polyphenol standards (malvidin-3-O-glucoside, naringin, catechin, quercetin, gallic acid, vanillic acid, caffeic acid, ferulic acid), amino acid standard mixture, methanol, acetic acid, gallic acid, were purchased from Merck (Darmstadt, Germany); 2-propanol and acetonitrile (ACN) from Honeywell (Charlotte, USA), formic acid by J.T. Baker (Rodano, Italy).

### 2.2 Plant material

**Figure 1:**
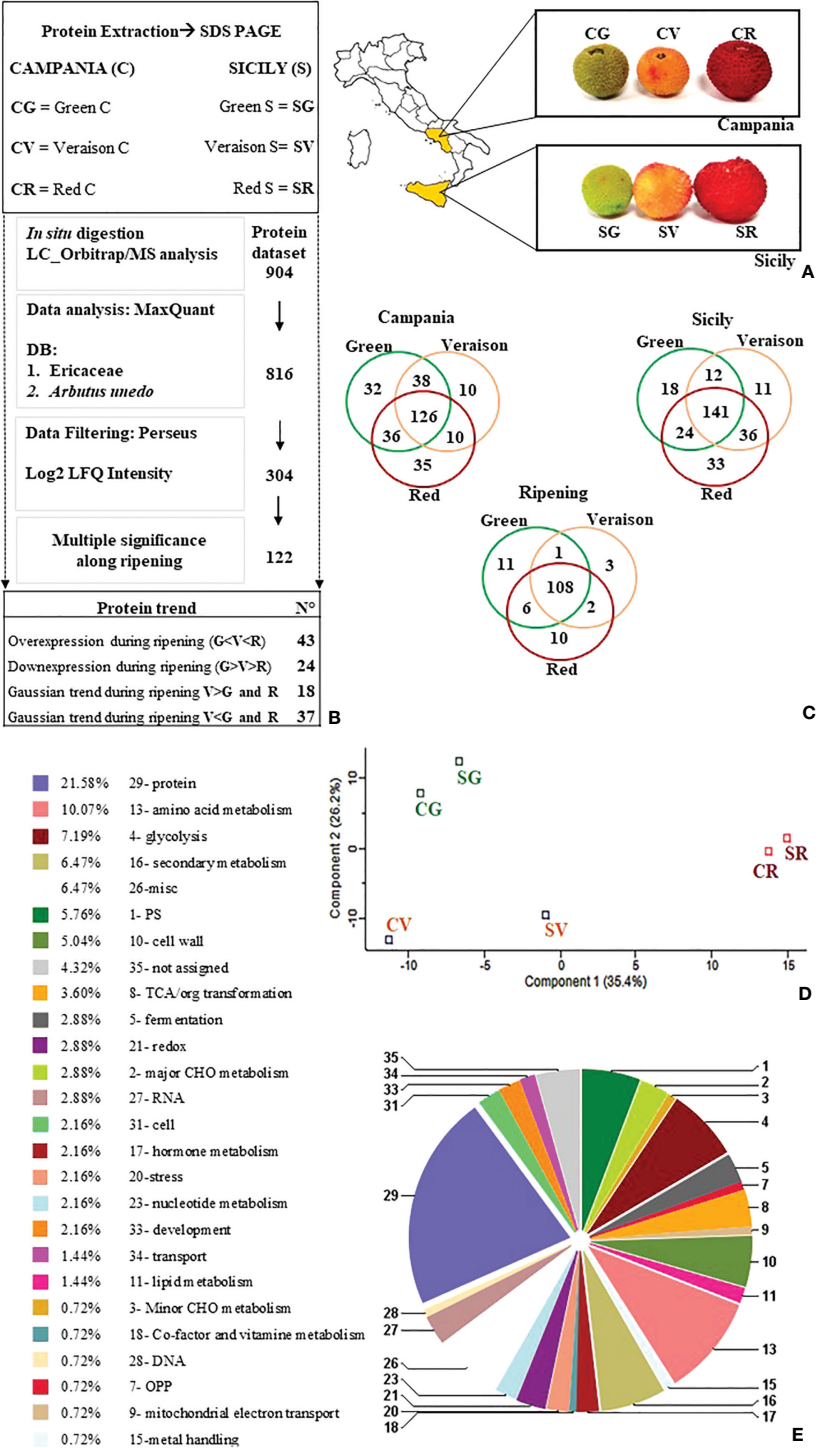
**(A)** *A. unedo* fruits in different ripening stages (G:green, V:veraison, R:red) sampled from two Italian regions, Sicily (S) and Campania (C). **(B)** Proteomic workflow followed. **(C)** Venn diagram related to the number of proteins quantified in green, veraison, and red fruits from Campania and Sicilia. The Ripening Venn diagram was obtained by averaging the data for both datasets along the ripening stages. **(D)** PCA analysis was performed to summarize and visualize the six entire datasets containing individuals/observations described by multiple inter-correlated quantitative proteins as variables. The plot explained 35.4% of component 1 and 26.2% of component 1 showing clustering of Campania and Sicily fruit samples (green, veraison, and red) collected in specific areas accordingly to the ripening stage. **(E)** Proteins differentially expressed by percentage abundance of functional classes by Mercator4 V2.0(https://mapman.gabipd.org/app/mercator).

*Arbutus unedo* fruits and leaves were collected at two sites, the first in Campania (C) (41°0’2.80’’ N, 14°46’55.92’’ E) and the second in Sicily (S) (37°51′11″41 N, 14°3′23″57 E). The climate of the collection area in Campania is typically Mediterranean, with average minimum temperatures of 8.0°C in winter and 23.8°C in summer; average annual rainfall, mostly distributed in the autumn/winter months, is around 900 mm (Aquino et al., 2008). The plants selected for collection in Campania are planted in deep, slightly alkaline and well-drained soil (Eutric Cambisol soil type). The second sampling site was located in the territory of Gratteri, a municipality in the metropolitan city of Palermo in Sicily. Gratteri falls within the Madonie Park (a short mountain ridge) (Catalano et al., 2013). The territory lies at an altitude of 400-1000 m. Soils of a carbonate and terrigenous nature predominate in the area. The sampling area is predominantly characterized by Regosol on clay rocks. Organic matter content is low, as are fertility elements in general (Maetzke et al., 2008). Sampling was carried out by collecting fruits and leaves on three trees of the species, to minimize intraspecific variability, the trees selected for sampling were of comparable size and age (approximately 30 years). The simultaneous presence of leaves and fruit in the 3 stages of ripening on the A.unedo tree made it possible to collect all the matrices under analysis at the same time. Fruits were randomly sampled from the selected trees at three stages of ripening: G (fruit fully developed but still totally green), V (fruit in veraison with a colour change from yellow to orange, flesh still firm), R (fruit fully ripe with soft flesh, totally red) (**Figure 1A**). Approximately 50 grams of leaves and 50 grams of fruit for every stage of ripening were sampled from each of the 3 selected trees in each geographical area. Samples of *A. unedo* were vacuum sealed directly into clean polyethylene bags and stored in refrigerated boxes, brought to the laboratory, thoroughly washed with distilled water to remove impurities (dust and small insects), dried and stored at -80°C until subsequent analyses.

### 2.3 Proteomics analysis methods

#### 2.3.1 Protein extraction

Total protein extraction was performed in duplicate from a composite sample obtained by sampling three fruits for each ripening stage/sampling site (**Figure 1A**), pounded in liquid nitrogen using 1% polyvinylpyrrolidone (PVP) per dry weight of sample. One gram of pulverised sample was suspended in 5 mL of extraction buffer (500 mM Tris-HCl, pH 7.5, 700 mM sucrose, 100 mM KCl, 50 mM EDTA, 2% w/v β-mercaptoethanol and 1 mM PMSF) for 15 min at 4°C, 5 mL of Tris-saturated phenol (500 mM Tris-HCl, pH 7.5) was added, stirred 10 min at 4°C and then centrifuged (15 min, 10,000g, 4°C). The proteins were precipitated in 5 volumes of ammonium acetate saturated in methanol for 20 min at -80°C and centrifuged (30 min, 10,000g, 4°C). The resulting protein pellet underwent three successive washes, the first in cold methanol and twice with cold acetone, dried and solubilised in a solubilisation buffer (7M urea, 2M thiourea, 4% (w/v) CHAPS, 40 mM DTT and 0.5% IPG buffer) for 1 hour at room temperature and centrifuged to remove insoluble material (1 min, 12,000g, 4°C). The protein concentration was estimated by the Bradford method, using bovine serum albumin as standard.

#### 2.3.2 Electrophoresis (SDS-PAGE)

The solubilised protein extracts, according to Laemmli et al. (Laemmli, 1970), were brought to 90°C in the SDS loading buffer and loaded onto 12% SDS-PAGE gels. The electrophoretic run included an initial 30-min phase at 60 V followed by approximately 2 h at 130 V in 1× Tris/Tricine/SDS buffer (Bio-Rad). The gel was stained with Coomassie Stain (EzBlueRstain Sigma-Aldrich reagent) and bleached overnight in 30% methanol and 10% acetic acid in water.

#### 2.3.3 *In situ* hydrolysis

Horizontal slices of SDS-PAGE bands for each sample (green, veraison, and red fruit from both Sicilia and Campania regions) were excised from the gel lane. Gel destaining consisted of the three consecutive cycles of 0.1 M NH_4_HCO_3_ at pH 8.0 and ACN, followed by reduction (10 mM DTT in 100 mM NH_4_HCO_3_, 45 min, and 56°C) and alkylation (55 mM IAM in 100 mM NH_4_HCO_3_, 30 min, and RT). The gel pieces were washed with three further cycles of 100 mM NH_4_HCO_3_ of pH 8.0 and ACN. Finally, the gel pieces were subjected to an enzymatic hydrolysis by covering them with 40 μL sequencing grade modified trypsin (10 ng·μL^–1^ trypsin; 10 mM NH_4_HCO_3_) and incubated overnight at 37°C. Peptide mixtures were eluted, vacuum-dried, and resuspended in 2% ACN acidified with 0.1% formic acid (HCOOH) before the LC-MS/MS (liquid chromatography-mass spectrometry and liquid chromatography-tandem mass spectrometry).

#### 2.3.4 LC-Orbitrap/MS analysis

The peptide mixtures were injected into an LTQ Orbitrap XL (ThermoScientific, Waltham, MA) coupled to a nano-LC system (nanoEasy II). A volume of 3 µL of each sample was loaded onto a C18 capillary reverse-phase column (100 mm, 75 μm, 5 μm) working at a 250 nL·min^–1^ flow rate, using a linear gradient of eluent B (0.2% formic acid in 95% ACN) in A (0.2% formic acid and 2% ACN in MilliQ water) from 5% to 40% in 80 minutes was run. MS/MS analyses were performed using Data-Dependent Acquisition (DDA) mode: one MS scan (mass range from 400 to 1800 m/z) was followed by MS/MS scans of the five most abundant ions in each MS scan, applying a dynamic exclusion window of 40 seconds.

#### 2.3.5 Proteomics data analysis

Raw data files were processed by using MaxQuant software (1.6.8.0 version) (Tyanova et al., 2015). An experimental design template was used to specify individual or merged replicate experiments (each data set contained two technical replicates) and to combine all raw data from each lane into a single column containing all the proteins in every sample. The following parameters were used for raw data processing: trypsin enzyme specificity, 3 missed tryptic cleavages, oxidation of methionine, and formation of pyroGlu from N-terminal glutamine (Q) or glutamic acid (E), as variable modifications, and cysteine (C) carbamidomethylation as a fixed modification. Identification parameters included a minimum peptide length of 6 amino acids, minimum of 1 peptide (both razor and unique peptide). Peptide tolerance of 10 ppm, fragment mass tolerance of ± 0.2 Da. All proteins were filtered according to a false discovery rate (FDR) of 0.01% applied both at peptide and protein levels and a maximum peptide posterior error probability (PEP) of 1. The derived peak list generated by Quant.exe (the first part of MaxQuant) was searched using the Andromeda search engine integrated into the MaxQuant both against *A. unedo* and Ericaceae fasta file downloaded from the UNIPROT web site. The MaxQuant file (protein.txt) was further uploaded on Perseus software (Tyanova et al., 2016) to perform the statistical analysis. Contaminants, reverse, and only identified by site hits were filtered out. Expression values of LFQ intensity were log_2_ transformed and only the protein rows containing a minimum of 2 valid values were maintained within the final matrix. Multi-scattering, hierarchical clustering heatmap, and PCA analysis were performed by using Perseus Finally, multiple significance test was performed by Perseus to obtain the dataset of significant proteins.

### 2.4 Metabolomics analysis methods

#### 2.4.1 Metabolites extraction

A triplicate maceration extraction (ME)(Scarano et al., 2020; Srivastava et al., 2021; Scarano et al., 2022) was performed from leaf and fruit samples with a ethanol (EtOH) 70%. Extractions were conducted at room temperature (25.00 ± 1.00°C). Between 5.0-6.5 g of leaf and fruit samples were extracted by maceration with 50-65 mL of EtOH 70% under continuous stirring and in the dark for 24 h. At the end of the maceration process, the extract sample was filtered on filter paper to remove any suspended matter to obtain a clear solution. The extract obtained was stored in a dark container to exclude the bioactive compounds photodegradation.

#### 2.4.2 Extracts purification

To purify the target compounds obtained a liquid-liquid extraction protocol was performed (Scarano et al., 2022). The extractions, performed with a separating funnel, involved the sequential use of three extraction solvents of increasing polarity (hexane, chloroform, and 1-butanol). All solutions containing the extracted samples were subjected to evaporation, using a HEIDOLPH Heizbad Hei-Vap rotary evaporator (Schwabach, Germany), to calculate the yield of each.

#### 2.4.3 Soluble solid contents

Soluble solid content (°Bx) was measured in all extracts by means of a refractometer: a Brix and Gravity Refractometer with automatic temperature compensation (ATC) (with detection range of 0-32% Brix Grade and 1.000-1.130 for Specific Gravity, respectively) was used for specific gravity detection.

#### 2.4.4 Total phenolic compound content (TPC)

The TPC content was measured according to the Folin-Ciocălteu reagent method (Dewanto et al., 2002), in which 50 μL of sample was added to a cuvette together with 2300 μL of double-distilled water and 50 μL of Folin-Ciocălteu reagent (reagent was diluted 1:2 in water solution). After 6 minutes, 100 μL of a sodium carbonate solution (Na_2_CO_3_) was added to the same cuvette. All cuvettes were shaken manually and allowed to stand for 90 min at room temperature (T = 25°C). Because, in some cases, the addition of sodium carbonate produces turbidity, which may result in an increase in the absorbance signal, the solution was filtered before measurement. The absorbance was measured at 760 nm, and the total content of phenolic compounds was expressed as gallic acid equivalents (GAE) as the concentration of GAE expressed in mol·L^-1^ using the calibration curve of gallic acid standard solutions (50-250 mg·L ^-1^). All measurements were taken in triplicate and calculated as mean value ± SD (n = 3). The absorbance measurements for TPC and for DPPH assay analysis were performed by a MERCK (Milano, Italy) Spectroquant^®^ Pharo 300 UV/Vis spectrophotometer, using a 1.0 cm long optical path glass cell.

#### 2.4.5 Scavenging activity for 1,1-diphenyl-2-picrylhydrazyl (DPPH) radical

The extracts scavenging activity was estimated using the DPPH assay according to Scarano et al., 2022 (Scarano et al., 2022). Absorption of the samples at 517 nm was determined spectrophotometrically at different time points (t0 to t9) over a ten-minute period with readings every minute. The radical scavenging activity was then expressed as the percentage of free radical inhibition by the sample and was calculated using the equation:


(1) $$\%DPPH\;radical\;scavenging\;activity=\left[\frac{\left(A_{control}-A_{sample}\right)}{A_{control}}\right]\times 100$$

where *A*_sample_ and *A*_control_ are the absorbance of the sample and the absorbance of the control solutions, respectively. The results were expressed as % free radical inhibition (I%). The I% is directly related to the antioxidant power of the sample.

#### 2.4.6 Targeted LC-MS/MS analysis

Mass spectrometry in multiple reaction monitoring ion mode (MRM) was used to identify and quantify target molecules both polyphenols and amino acids. A 4000 QTRAP from AB Sciex (Darmstadt/Germany), equipped with an ESI source and a hybrid triple quadrupole–LIT (linear ion trap) was used to perform the mass spectrometry analyses. The analyses were performed both in positive and negative ion mode. Source dependent parameters like curtain gas (CUR), collision gas (CAD), ion spray voltage (IS) and source temperature (TEM) were set at 20.0 psi, 5 psi, 4.5 kV, 380.0°C respectively. Liquid chromatography was performed on an LC Eksigent operating with a column Halo C18 2.7 µm 90A 1*50 mm (Munich, Germany) at a temperature of 45°C. The elution was performed during a total run of 8.5 min at a flow rate of 40 µL/min using a mobile phase A containing 0.1% formic acid, 5 mM ammonium formate in water and a mobile phase B consisting of 0.1% formic acid, 5 mM ammonium formate in ACN (B) as a mobile phase. The gradient table for LC run provided the following gradient: 0-1 min at 1% B; 1-3 min at 12% B; 3-6 min at 20% B; 6-8 min at 99% B; 8-8.5 min at 1%B. The MRM/MS methods containing the target molecules and related precursor and product ions (m/z) were previously published (Pinto et al., 2021; Illiano et al., 2022) and an application of this methodology on *A. unedo* has been recently reported (Scarano et al., 2022). Quantitative analyses were performed by using the external standard method analogously reported by Scarano et al., 2022 (Scarano et al., 2022). Data interpretation was realized by Skyline software 20.2.0.343 version (MacCoss Lab, Department of Genome Sciences, UW) by importing the.wiff files obtained from instrumental analyses. The Skyline software was used to process the multi-replicate data, the chromatographic peaks corresponding to each compound were identified and the peak areas were extracted and interpolated on calibration curves, accordingly to structural homology, to obtain the compound concentration expressed as ng·g^-1^ of fruit weight.

#### 2.4.7 Metabolomics data analysis

The graph of individuals from the output of Principal Component Analysis (PCA) was performed in R environment using factoextra package (Kassambara and Mundt, 2020). Principal component analysis (PCA) was adopted to reduce the dimensionality of multivariate data to two components that can be visualized graphically with minimal loss of information. The graph of individuals showed similar specimen grouped together on the plot.

## 3 Results

### 3.1 Proteomic profiles of *A. unedo* fruits under the ripening process in different Italian locations

In the current shotgun proteomics experiment, proteins extracted from green, veraison, and red *A. unedo* fruits, from two Italian regions (*e.g.* Sicily and Campania **Figure 1A**), were separated by SDS-PAGE, and all bands excised from the entire lane. Each band was subjected to a classic protocol of *in situ* digestion and protein mixture analysed by LC-MS/MS. Next, MS/MS data acquired from the analyses were processed by using MaxQuant, visualized, and filtered by Perseus software. The list of 304 selected proteins was included in the **supplementary material** (**Supplementary File 1**). The 304 proteins were represented by Venn diagrams for the Campania and Sicilia individually taken, and for the one obtained by merging them, to figure out the expression of proteins along the ripening stage (**Figure 1B**). The Venn diagrams highlighted how most of proteins (126, 141, and 108) were shared between all the samples, regardless of region or maturation stage (**Figure 1C**). The higher number of exclusive proteins was mainly quantified in red fruits followed by that in green and veraison fruits (**Figure 1C**). On the 304-protein list a PCA analysis were performed, by using Perseus to visualize the data correlation among all samples. The PCA results showed a clear separation of samples according to ripeness stage (CG-SG/CV-SV/CR-SR) rather than sampling region (**Figure 1D**). To carry out a statistical analysis of the ripening stage of fruits, the log_2_ LFQ intensities (including those replaced by random numbers) of each protein quantified in green, veraison, and red Campania fruits were averaged with those from Sicily (see material and method section). The Campania and Sicilia proteins lists were grouped according to the ripening stage (green, veraison, and red) to figure out the differentially abundant proteins along with the maturation, regardless of the origin region. At this aim, missing values were replaced by random numbers drawn from a normal distribution with a width of 0.3 and a downshift of 1.8 as suggested by other authors (Hediyeh-zadeh et al., 2020). A multidimensional significance test was finally performed to extract a list (122 proteins) containing only the statistically significant proteins (Benjamini-Hochberg false discovery rate (FDR) ≤ 0.05) in two or more ripening stages. The list of 122 proteins was included in the supplementary material (**Supplementary File S2**). A multi-significance analysis performed by Perseus picked out a smaller dataset (122 proteins) (see **Table S2**) with changes of expression resulting to be statistically significant (P-value<0.05). These proteins were divided according to their functional class using Mercator4 V2.0 (https://mapman.gabipd.org/app/mercator) as shown in **Figure 1E**. The functional classes with the highest representation were involved in protein metabolism (21.58%), amino acid metabolism (10.07%), glycolysis (7.19%), secondary metabolism (6.47%), photosynthetic processes (5.76%) and cell wall (5.04%).

**Figure 2:**
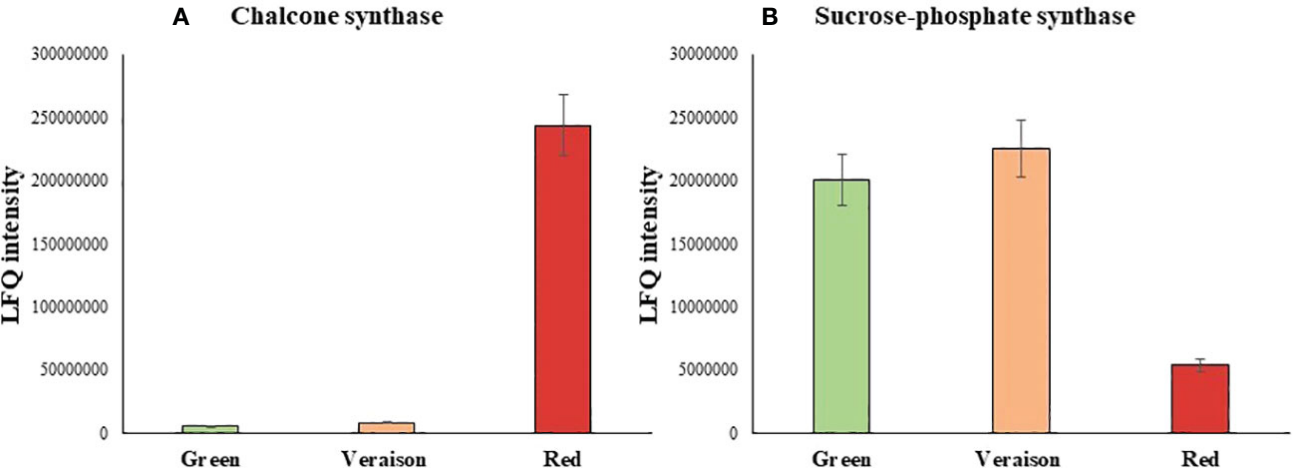
Histogram of LFQ intensity recorded for each sample and grouped in agreement with the trend of expression of Chalcone synthase **(A)** and Sucrose-phosphate synthase **(B)** in Green, Veraison and Red *A.unedo* fruits.

Among the differentially abundant proteins, a marked protein overexpression during ripening (G<V<R) characterized 43 proteins, including 3 proteins displaying fold changes in red fruit of 45-30 higher than in green. The highest fold change (red *versus* green) was recorded for the chalcone synthase, a key enzyme involved in the flavonoid/isoflavonoid biosynthesis pathway through the condensation of one p-coumaroyl and three malonyl-CoA molecules to form naringenin. This enzyme has been demonstrated to be responsible for the red colour of the fruit such as for ripe tomato berry by accumulating flavonoids in the cuticle of the fruit (España et al., 2014). Interestingly, the overexpression was particularly marked during the last step of ripening as demonstrated by the low ratio of protein expression between green and veraison (1.6-fold change). A high level of sucrose-phosphate synthases, an enzyme involved in the sucrose biosynthesis, was mainly observed in green and veraison fruits with a reduction of 4-fold in those red suggesting a decrease of sucrose in ripened fruit. Then, a total of 24 proteins were downregulated along with the ripening with variable ratios of protein expression between red or green fruits with veraison one (**Figure 2**).

Among the 55 proteins displaying a gaussian trend, roughly 30% of proteins showed an upregulation in veraison fruits, and almost 20% were involved in the amino acid biosynthesis suggesting substantial changes in amino acid levels during this ripening stage (**Supplementary File 2**). Interestingly, as detailed later, the synthesis of four amino acids was mainly dysregulated during the fruit ripening. Indeed, phenylalanine and isoleucine were upregulated in veraison fruits whereas aspartate and tryptophan were downregulated.

### 3.2 Metabolic profiles of *A. unedo*

Metabolomic analysis of *A. unedo* fruits at different stages of ripening (green, veraison and red), and leaves, sampled in different geographical areas of the Italian peninsula (Campania and Sicily), were carried out to obtain a broad characterisation of this species at risk of cultural erosion.

#### 3.2.1 Soluble solid contents

The extraction protocol performance was evaluated through the quantification of the solvent extractive capacity against the plant materials. The values were showed in **Table 1**.

**Table 1:** Specific gravity, dry residue and extract value of the different *A. unedo* extracts.

Sample	*Code*	*Brix*	*Residue*	*Extract*
		[°Bx]	(g·L^-1^)	(g·g^-1^ F.W.)
***Leaf Campania***	**CL**	1.1 ± 0.1	7.62 ± 0.27	0.0882 ± 0.0017
***Leaf Sicilia***	**SL**	1.0 ± 0.1	11.16 ± 0.28	0.1305 ± 0.0054
***Green fruit Campania***	**CG**	1.3 ± 0.1	17.48 ± 0.02	0.1774 ± 0.0024
***Veraison fruit Campania***	**CV**	1.4 ± 0.2	45.95 ± 0.22 ^*^	0.4565 ± 0.0024 ^*^
***Red fruit Campania***	**CR**	1.5 ± 0.2	58.12 ± 0.12 ^*^	0.5806 ± 0.0061 ^*^
***Green fruit Sicilia***	**SG**	1.2 ± 0.1	19.04 ± 0.15 ^*^	0.1815 ± 0.0018 ^*^
***Veraison fruit Sicilia***	**SV**	1.1 ± 0.1	51.67 ± 0.11 ^*^	0.4887 ± 0.0021 ^*^
***Red fruit Sicilia***	**SR**	1.3 ± 0.1	63.28 ± 0.26 ^*^	0.6230 ± 0.0019 ^*^

Values are presented as mean (triplicate) ± SD.*, Asterisks indicate significant differences for parameters between samples at p< 0.05. F.W., fresh weight.

As can be seen from the data showed in **Table 1**, there were slight differences between samples of different origins, both in terms of dry residue and total extract obtained; however, no significant differences were observed in Bx°. Evaluation of the soluble solid (expressed in degrees Brix, °Bx) clarified how in quantifying extracts, considering their specific gravity (expressed in **Table 1** as in grams of extract per grams of fresh sample weight), this value was very low and did not represent, in terms of weight, the majority component.

#### 3.2.2 Total phenolic compound (TPC) and radical scavenging activity

All fractions into which extracts were quantified were subjected to preliminary analysis: total phenolic content by Folin-Ciocălteu reagent and scavenging activity by the DPPH method and showed in **Table 2**. Only the 1-butanol fractions were found to be the richest in polyphenols and antioxidant compounds, so they were used to perform subsequent molecular characterization of A. unedo leaves and fruits.

**Table 2:** Total phenolic compound content (TPC) and DPPH radical scavenging activity (I%) of the different *A. unedo* extracts.

Sample	Code	Total phenolic compound content (*C* GAE·(mol·L^−1^))	% Free radical inhibition (I%)
***Leaf Campania***	**LC**	0.1943 ± 0.0061	21.30 ± 0.42
***Leaf Sicilia***	**LS**	0.2226 ± 0.0049 ^*^	38.76 ± 6.06 ^*^
***Green fruit Campania***	**CG**	0.3553 ± 0.0090	57.14 ± 17.14
***Veraison fruit Campania***	**CV**	0.3589 ± 0.019	60.86 ± 1.41
***Red fruit Campania***	**CR**	0.3670 ± 0.0019	61.81 ± 0.59
***Green fruit Sicilia***	**SG**	0.3781 ± 0.0074	53.43 ± 7.43
***Veraison fruit Sicilia***	**SV**	0.3818 ± 0.0057	68.37 ± 3.91 ^*^
***Red fruit Sicilia***	**SR**	0.4104 ± 0.0089 ^*^	69.08 ± 4.28 ^*^

Values were presented as mean (triplicate) ± SD. GAE expresses the Equivalents of Gallic Acid. Asterisks indicate significant differences for parameters between the two extraction methods at p< 0.05.

The data for TPC and I% showed a difference between samples of different origins with a slight preference in the Sicilians *A. unedo* leaves samples. In comparison, in the fruit, although no significant differences were observed between the samples from the two sampling areas, significant accumulation of phenolic compounds and radical scavenging activity was observed during the ripening stages.

#### 3.2.3 LC-MS/MS analysis of *A. unedo* extracts

**Figure 3:**
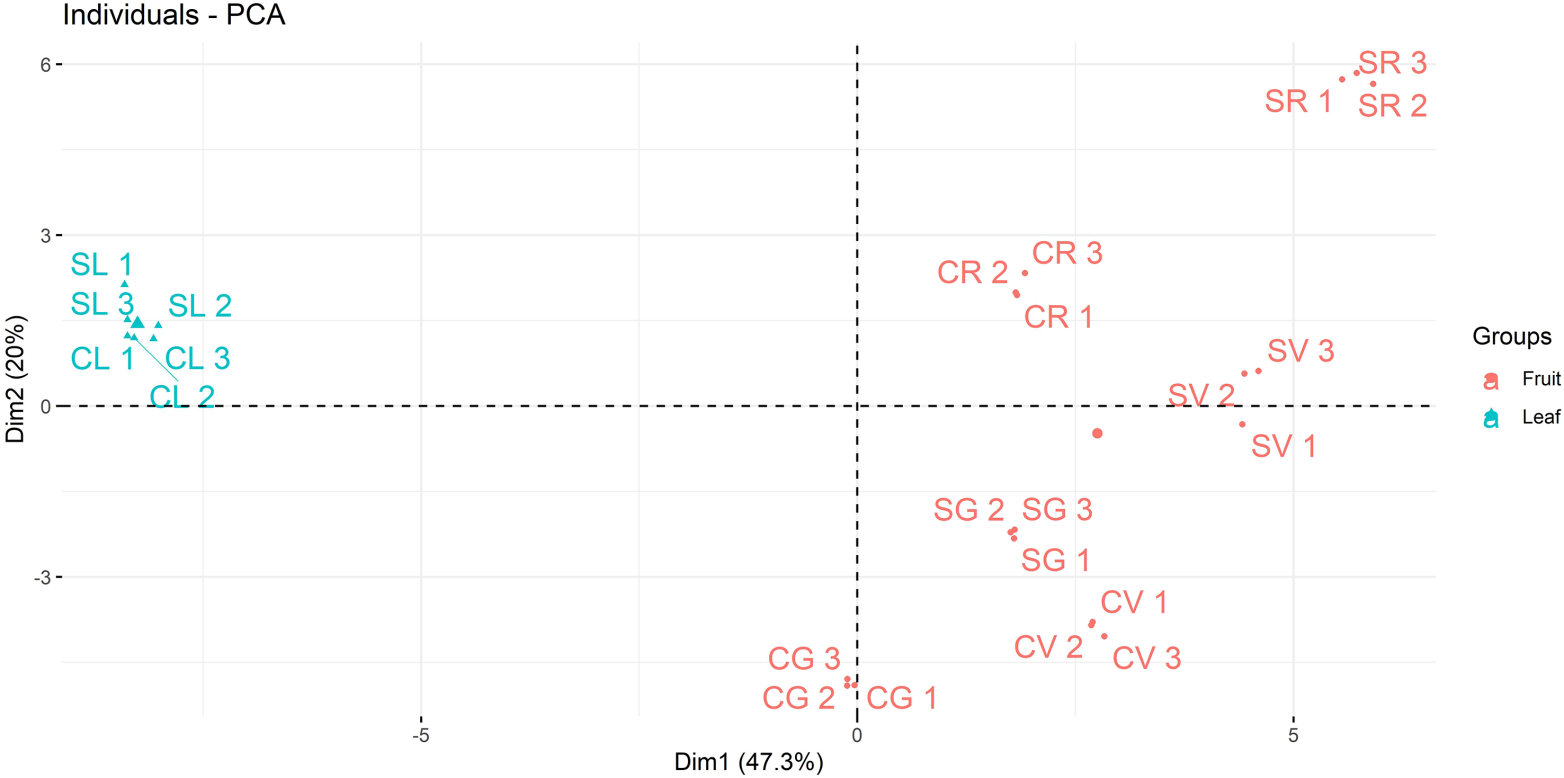
PCA analysis of polyphenols in green (G), veraison (V) and red (R) *A. unedo* fruit and leaves (L) from Campania (C) and Sicily (S) regions.

The polyphenolic fraction of *A. unedo* leaves and fruits samples from different geographical areas of the Italian peninsula, *e.g.* Campania and Sicily, and at different ripening stages (green, veraison and red) was characterized by using a targeted mass spectrometry-based approach. The results of the LC-MS/MS analysis in MRM ion mode were reported in **Table 3** and **Supplementary File 3**. A good clusterisation of leaves and fruits for polyphenols suggested a similar composition of such molecules in both Campania and Sicily samples whereas the red fruit was quite different from the others. Moreover, a different distribution of polyphenols was observed between the two regions within the PCA biplot displaying a differential polyphenols profile perhaps due to the response of fruit to the specific territorial conditions. As expected from the analysis of two different organs (leaf/fruit), a clear separation was observed between leaf and fruit samples at different ripening stages (**Figure 3**).

**Table 3:** Polyphenols identified in *A. unedo* fruit at different ripening stages and in *A. unedo* leaves by LC-MS analysis (expressed as ng/g of fresh weight, ng/g FW) extracts by LC-MS/MS analysis (expressed in ng·g^-1^ of F.W.

Antocyanins	CG	CV	CR	SG	SV	SR	CL	SL
**delphinidin-3-O-glucoside**	2053.02 ± 8.37	732.2 ± 7.71	1066.52 ± 7.03	3640.04 ± 63.23	1343.86 ± 29.69	2334.87 ± 28.30	15.47 ± 2.96	22.02 ± 6.62
**delphinidin-3-O-arabinoside**	812.01 ± 8.20	453.88 ± 11.01	759.65 ± 17.33	1477.61 ± 66.78	741.98 ± 22.08	1366.32 ± 32.49	346.72 ± 5.28	684.52 ± 18.27
**delphinidin rutinoside**	162.25 ± 8.44	44.96 ± 2.76	56.06 ± 12.06	337.53 ± 1.91	81.61 ± 4.80	98.57 ± 5.90	44.70 ± 3.01	418.63 ± 8.67
**delphinidin diglucoside**	81.16 ± 8.48	57.42 ± 4.09	165.49 ± 4.90	161.21 ± 7.46	174.56 ± 29.89	318.01 ± 16.05	6.28 ± 0.25	9.28 ± 2.07
**cyanidin-3-O-glucoside**	176.31 ± 1.15	326.98 ± 31.24	4902.21 ± 28.78	358.82 ± 23.61	2137.79 ± 55.65	9534.52 ± 54.75	8.16 ± 4.06	11.26 ± 2.02
**cyanidin-3-O-arabinoside**	123.33 ± 5.26	39.1 ± 3.97	1155.30 ± 38.50	255.00 ± 15.01	353.52 ± 45.93	2043.94 ± 21.06	61.44 ± 5.75	488.08 ± 2.71
**cyanidin-3-5-di-O-glucoside**	6.70 ± 0.20	13.09 ± 1.14	794.89 ± 5.07	35.65 ± 3.32	217.63 ± 4.65	1536.43 ± 41.71	4.94 ± 0.34	6.60 ± 1.28
**malvidin-3-O-glucoside**	222.07 ± 2.62	50.57 ± 3.42	57.00 ± 7.23	453.58 ± 21.15	187.04 ± 28.05	104.19 ± 4.75	19.80 ± 11.80	9.67 ± 1.33
**malvidin-3-O-arabinoside**	55.46 ± 5.11	9.35 ± 0.67	7.90 ± 0.45	108.69 ± 6.09	44.366 ± 47.80	16.42 ± 1.33	11.39 ± 6.25	8.57 ± 0.99
**malvidin-3-O-p-coumaroylglucoside**	4.52 ± 0.03	9.51 ± 0.10	18.35 ± 0.32	10.14 ± 0.27	30.779 ± 12.34	34.78 ± 1.00	5.14 ± 0.28	5.19 ± 0.16
**petunidin-3-O-glucoside**	15.08 ± 1.11	86.66 ± 1.06	71.04 ± 5.08	113.34 ± 0.87	175.32 ± 11.72	137.99 ± 9.85	6.78 ± 0.43	27.86 ± 3.97
**petunidin-3-O-arabinoside**	4.24 ± 0.42	38.91 ± 3.26	66.64 ± 2.96	29.61 ± 2.28	63.08 ± 13.90	161.36 ± 12.89	n.q ± 0.00	n.q ± 0.00
**peonidin-3-O-glucoside**	18.20 ± 0.50	258.64 ± 19.00	180.74 ± 16.85	95.37 ± 6.45	347.61 ± 19.90	382.36 ± 17.27	n.q ± 0.00	n.q ± 0.00
**pelargonidin-3-O-glucoside**	67.19 ± 1.77	141.22 ± 6.18	149.22 ± 15.87	159.88 ± 7.62	315.42 ± 18.86	347.05 ± 28.00	7.71 ± 2.99	8.45 ± 0.55
Catechins								
**catechin**	3089.11 ± 16.69	1564.97 ± 24.72	37339.18 ± 34.10	5732.98 ± 25.65	2604.36 ± 70.02	42354.69 ± 76.40	3645.84 ± 49.06	2614.80 ± 29.01
**procyanidin B1**	316.88 ± 2.89	75.18 ± 2.62	432.03 ± 17.65	472.08 ± 12.21	242.83 ± 31.74	745.59 ± 27.44	62.043 ± 1.51	61.35 ± 12.09
**procyanidin C**	11.38 ± 0.84	23.02 ± 3.46	33.22 ± 3.86	41.83 ± 3.99	51.55 ± 1.90	56.79 ± 4.82	4.00 ± 0.99	35.25 ± 10.02
**procyanidin tetramer**	10.88 ± 0.76	50.98 ± 4.65	82.45 ± 4.48	85.58 ± 5.50	90.09 ± 5.07	152.88 ± 7.93	1.10 ± 1.04	5.37 ± 3.32
**procyanidin pentamer**	n.q. ± 0.00	n.q. ± 0.00	n.q. ± 0.00	n.q. ± 0.00	n.q. ± 0.00	n.q. ± 0.00	0.92 ± 0.72	2.43 ± 2.00
**catechin-3-gallate**	105.35 ± 7.38	190.59 ± 6.43	114.05 ± 8.51	139.10 ± 3.92	127.66 ± 6.89	117.47 ± 20.94	10.95 ± 2.55	59.39 ± 1.65
**gallocatechin**	165.83 ± 5.26	241.16 ± 11.87	474.19 ± 17.75	179.28 ± 4.65	287.64 ± 37.93	213.70 ± 20.29	917.10 ± 5.66	753.47 ± 18.82
**EC-3-gallate**	145.23 ± 8.91	180.15 ± 13.26	115.62 ± 7.84	142.05 ± 6.89	175.65 ± 11.43	122.41 ± 11.38	10.95 ± 2.55	78.90 ± 9.60
**EGC 3-gallate**	114.51 ± 3.38	96.803 ± 6.43	70.35 ± 4.96	130.58 ± 6.51	125.63 ± 29.13	128.40 ± 12.18	126.70 ± 7.19	13.016 ± 3.28
**GC 3-gallate**	116.47 ± 12.21	156.57 ± 3.38	63.04 ± 14.39	251.98 ± 11.22	120.94 ± 25.95	129.45 ± 6.71	11.10 ± 1.99	13.016 ± 3.31
**EGC-epicatechin dimer**	23.95 ± 0.44	23.61 ± 1.49	71.93 ± 6.38	42.25 ± 2.67	37.38 ± 5.14	179.02 ± 4.11	62.89 ± 4.74	108.28 ± 6.90
**EGC digallate**	7.02 ± 0.49	10.98 ± 0.86	55.20 ± 2.44	19.17 ± 1.33	35.75 ± 3.15	90.79 ± 6.00	9.43 ± 1.75	22.78 ± 11.62
**EGC gallate glucoside**	27.33 ± 1.33	37.86 ± 2.84	32.75 ± 7.67	12.82 ± 0.37	32.40 ± 3.40	29.20 ± 2.94	9.34 ± 1.38	12.22 ± 0.47
**EGC-EGC gallate**	n.q. ± 0.00	n.q. ± 0.00	n.q. ± 0.00	n.q. ± 0.00	n.q. ± 0.00	n.q. ± 0.00	5.88 ± 2.50	7.38 ± 0.17
Polyphenols								
**flavone+Na**	296.47 ± 15.60	413.54 ± 20.83	158.64 ± 15.04	146.81 ± 10.42	258.25 ± 30.55	79.58 ± 4.51	15.33 ± 9.75	5.56 ± 0.11
**apigenin**	637.57 ± 6.31	966.30 ± 10.71	783.73 ± 32.15	420.57 ± 22.65	413.38 ± 57.07	426.80 ± 20.97	16.34 ± 8.93	9.18 ± 4.54
**daidzein**	194.62 ± 15.46	266.25 ± 15.69	38.22 ± 6.41	382.52 ± 27.98	357.09 ± 15.19	292.32 ± 33.46	7.88 ± 1.44	8.26 ± 0.91
**eriodictyol**	299.49 ± 33.30	1306.79 ± 17.62	1823.40 ± 38.55	1024.41 ± 38.10	1306.33 ± 54.84	1705.89 ± 183.88	65.50 ± 10.91	30.11 ± 7.13
**quercetin**	4495.07 ± 7.19	2253.10 ± 26.84	4636.60 ± 35.56	2819.72 ± 52.54	3300.54 ± 41.87	4704.42 ± 112.77	104.79 ± 2.89	165.88 ± 3.03
**quercetin-3-glucoside**	1934.26 ± 17.95	767.17 ± 55.21	891.19 ± 22.53	857.71 ± 41.50	733.53 ± 79.09	690.93 ± 41.00	154.10 ± 11.87	1323.99 ± 34.97
**sinensetin**	318.65 ± 16.39	107.33 ± 13.02	65.00 ± 6.59	n.q. ± 0.00	128.63 ± 7.98	134.83 ± 22.28	7.082 ± 1.43	4.54 ± 0.00
**naringin**	241.17 ± 18.67	223.26 ± 15.07	255.19 ± 23.25	274.39 ± 19.21	353.59 ± 14.09	294.53 ± 21.68	34.76 ± 3.18	11.30 ± 5.64
**naringenin**	310.56 ± 6.26	931.68 ± 18.30	468.61 ± 16.99	484.49 ± 18.76	744.81 ± 46.99	513.60 ± 45.46	29.79 ± 0.66	16.47 ± 4.15
**myricetin**	16.64 ± 0.71	16.53 ± 0.90	10.23 ± 1.15	32.65 ± 1.80	27.26 ± 0.38	23.48 ± 3.71	n.q ± 0.00	n.q ± 0.00
**myricitrin**	417.66 ± 13.56	672.06 ± 32.28	838.55 ± 40.88	443.93 ± 22.94	1195.75 ± 89.73	907.66 ± 48.05	n.q ± 0.00	n.q ± 0.00
**kaempferol**	281.65 ± 14.59	390.25 ± 49.49	161.12 ± 13.71	311.86 ± 20.37	193.28 ± 22.18	167.43 ± 13.56	n.q ± 0.00	n.q ± 0.00
**phloridzin**	789.13 ± 8.01	1215.95 ± 30.10	1401.28 ± 90.57	698.16 ± 37.80	1176.45 ± 24.07	1095.52 ± 78.08	160.61 ± 10.28	15.73 ± 1.63
**phloretin**	176.49 ± 6.68	185.16 ± 3.24	146.82 ± 21.32	182.32 ± 14.58	80.61 ± 16.62	86.96 ± 11.93	48.32 ± 4.92	20.50 ± 12.17
**rutin**	144.52 ± 12.93	39.53 ± 3.48	56.20 ± 2.97	75.83 ± 4.69	20.97 ± 1.35	29.87 ± 1.68	32.55 ± 1.86	418.03 ± 17.29
**6-malonyldaidzin**	98.47 ± 5.27	153.89 ± 12.58	109.59 ± 6.83	53.31 ± 0.35	73.98 ± 7.51	58.46 ± 0.66	8.11 ± 2.19	6.58 ± 0.34
Phenolic acids								
**gallic acid**	11959.99 ± 45.56	9339.06 ± 10.12	3412.32 ± 6.63	217.51 ± 6.50	565.74 ± 28.88	460.45 ± 43.02	468.05 ± 18.35	447.26 ± 10.76
**syringic acid**	2196.21 ± 16.26	2129.88 ± 26.61	718.08 ± 29.17	765.37 ± 27.58	625.35 ± 14.49	801.32 ± 69.52	164.51 ± 9.93	163.58 ± 4.47
**chlorogenic acid**	270.69 ± 10.92	822.94 ± 18.36	409.11 ± 28.87	340.63 ± 26.98	577.74 ± 41.94	587.82 ± 22.95	176.25 ± 9.72	184.70 ± 5.13
**caffeic acid**	226.12 ± 6.65	195.48 ± 20.21	169.21 ± 19.99	303.73 ± 3.43	124.82 ± 30.31	153.27 ± 19.50	211.05 ± 12.43	249.47 ± 12.04
**quinic acid**	1168.47 ± 34.66	2748.38 ± 47.25	1106.42 ± 10.51	2282.99 ± 176.10	4793.55 ± 27.95	3348.62 ± 77.67	n.q ± 0.00	n.q ± 0.00
**ferulic acid**	1822.23 ± 21.00	2411.39 ± 41.35	819.62 ± 28.94	4104.09 ± 94.34	4501.72 ± 98.00	3869.20 ± 57.93	175.74 ± 13.12	190.20 ± 13.39
**coumaric acid**	132.30 ± 9.57	49.00 ± 1.46	n.q. ± 0.00	193.84 ± 14.58	86.96 ± 25.26	11.49 ± 1.01	209.06 ± 15.64	252.94 ± 5.31
**vanillic acid**	573.03 ± 36.79	364.52 ± 25.58	201.87 ± 0.62	578.93 ± 26.54	384.64 ± 24.85	383.03 ± 15.35	1046.86 ± 91.73	248.20 ± 18.03
**coumaroylquinic acid**	171.64 ± 7.05	193.09 ± 15.44	225.30 ± 14.35	171.06 ± 7.96	282.77 ± 5.63	202.60 ± 9.96	n.q ± 0.00	n.q ± 0.00
**feruloylquinic acid**	230.13 ± 3.65	177.25 ± 7.77	145.59 ± 15.62	206.99 ± 22.13	170.68 ± 7.41	174.82 ± 23.45	146.26 ± 2.52	147.21 ± 2.09
**valoneic acid dilactone**	19.02 ± 1.87	23.60 ± 2.83	14.12 ± 0.95	17.68 ± 1.15	36.00 ± 13.25	16.13 ± 2.09	n.q ± 0.00	n.q ± 0.00
**syringaldehyde**	n.q. ± 0.00	n.q. ± 0.00	n.q. ± 0.00	n.q. ± 0.00	n.q. ± 0.00	n.q. ± 0.00	292.39 ± 2.96	192.69 ± 6.62

Values for individual samples available in **Supplementary File 3**).

A generally higher total presence of anthocyanins can be observed in the Sicilian samples, especially in the green fruit. In detail, malvidin, petunidin, cyanidin and delphinidin coupled with glucose were more abundant than those containing arabinose or di-glycosidic forms. Cyanidin-3-glucoside increased with ripening, and the SR sample contained about twice the CR content. On the other hand, a downward trend was observed for the monitored malvidins, decreasing with ripening stages from green to red for both the Campanian and Sicilian samples. Very similar trends were observed in leaf samples from the two geographic areas. As for catechins, these showed an increase with ripening stages from green to red with peaks in abundance in CR and SR samples. A similar trend was also observed for the polymeric forms of catechin. Free quercetin was higher than the glycosylated form for each region and ripening stage, while significantly lower levels were found in leaf samples. Naringenin was higher than naringin for all samples and showed the highest concentration at the veraison stage. Similarly, myricitrin was much higher than myricitrin, with a higher concentration in the veraison samples than in the green and red ripening stages. Among the organic acids monitored, gallic acid was the most abundant, presenting highest concentrations in the Campania samples compared to the Sicilian samples. Quinic and ferulic acid presented the highest values in the veraison stage. Peculiar was the exclusive presence of syringaldehyde in leaf samples CL and SL (**Table 3**; **Supplementary File 3**).

To complement the proteomic analysis performed on the Campanian (CG, CV, CR) and Sicilian (SG, SV, SR) *A. unedo* fruit samples, the amino acid content was also analysed on the same samples. Amino acids were analysed by mass spectrometry by using the targeted approach and the results, reported as a percentage (%) in **Table 4**. Tryptophan and threonine, two essential amino acids, had the highest content in *A. unedo* fruit in particular, the former was much greater in the green ripening state than in the other ripening stages. Furthermore, for this amino acid the content in CG was higher than in SG. Threonine had a maximum concentration in the veraison stage for both Campania and Sicily. Valine, isoleucine and phenylalanine, essential amino acids, increased with ripening stages from green to red. Amino acids showing the maximum concentration in the veraison stage were lysine, histidine, methionine and the aforementioned threonine. On the other hand, asparagine tended to decrease along the fruit maturation displaying the highest value recorded for the sample of green Sicilian fruit (SG).

**Table 4:** Amino acids identified in *A. unedo* fruit from Campania and Sicily region at different ripening stages by LC-MS analysis (mean values expressed as percentage %, complete data available in **Supplementary File 4**).

Amino acid	CG	CV	CR	SG	SV	SR
**alanine**	1.34 ± 0.04	1.11 ± 0.01	0.58 ± 0.01	1.17 ± 0.02	0.00 ± 0.00	0.99 ± 0.02
valine*	0.56 ± 0.02	3.41 ± 0.14	5.72 ± 0.35	2.61 ± 0.04	3.18 ± 0.14	7.95 ± 0.13
leucine*	1.13 ± 0.08	5.43 ± 0.52	4.76 ± 0.15	5.23 ± 0.28	6.08 ± 0.67	6.10 ± 0.08
isoleucine*	7.69 ± 0.09	3.93 ± 0.16	10.11 ± 0.36	5.78 ± 0.01	4.58 ± 0.33	9.70 ± 0.07
tryptophan*	57.89 ± 2.98	4.83 ± 0.26	8.73 ± 0.23	41.64 ± 2.41	7.08 ± 1.04	9.99 ± 0.25
**tyrosine**	4.65 ± 0.22	11.37 ± 1.30	13.82 ± 1.20	5.38 ± 0.04	11.71 ± 1.03	14.99 ± 1.00
phenylalanine*	0.15 ± 0.01	3.31 ± 0.04	8.87 ± 0.61	1.09 ± 0.11	4.23 ± 0.32	8.04 ± 0.11
**aspartic acid**	2.45 ± 0.08	8.55 ± 0.26	3.07 ± 0.26	5.87 ± 0.07	6.93 ± 0.71	3.65 ± 0.21
**asparagine**	6.87 ± 0.18	8.33 ± 0.61	3.21 ± 0.16	12.41 ± 0.61	8.14 ± 0.44	3.49 ± 0.23
**glutamine**	0.07 ± 0.01	1.62 ± 0.11	1.11 ± 0.09	0.09 ± 0.01	1.35 ± 0.11	1.51 ± 0.12
**glutamic acid**	0.77 ± 0.04	9.45 ± 1.22	11.01 ± 0.18	1.13 ± 0.04	7.52 ± 0.34	9.40 ± 0.64
**arginine**	0.04 ± 0.00	1.33 ± 0.08	0.38 ± 0.02	0.06 ± 0.00	1.10 ± 0.07	0.67 ± 0.03
lysine*	0.12 ± 0.01	1.91 ± 0.09	2.04 ± 0.20	0.19 ± 0.01	1.86 ± 0.17	1.27 ± 0.06
histidine*	0.06 ± 0.00	2.73 ± 0.07	0.86 ± 0.05	0.39 ± 0.02	2.63 ± 0.16	1.01 ± 0.06
**serine**	0.59 ± 0.03	2.93 ± 0.09	1.68 ± 0.01	0.87 ± 0.08	3.21 ± 0.29	1.56 ± 0.03
threnonine*	14.21 ± 0.87	28.13 ± 2.46	23.36 ± 2.06	15.32 ± 0.76	29.15 ± 3.63	19.74 ± 0.46
methionine*	0.15 ± 0.00	1.65 ± 0.00	1.48 ± 0.00	0.22 ± 0.00	1.26 ± 0.00	1.08 ± 0.00

* stands for essential amino acids.

## 4 Discussion

The consumption of wild edible plants, which is closely related to a region’s cultural history, is part of people’s traditional and local identity, and is often due to the bioactive components and ethnopharmacological relevance rather than mere food value. However, the agricultural development, subject to the market logic, selected a limited number of plant species that were cultivated and commercialised, at the expense of less profitable plants that were unfortunately subject to cultural erosion (Bacchetta et al., 2016). The characterisation of such species could be a valuable enhancement strategy, especially if focused on bioactive molecules. For this purpose, this work focused on the traditionally consumed matrices (fruit and leaf) of *Arbutus unedo*, an underutilized fruit-tree typical of the Mediterranean region (Ruiz-Rodríguez et al., 2011). The characterization of protein abundance during the ripening process of *A. unedo* fruits revealed the implication of the main functional classes involved in the physiological changes typical of the ripening process and in the change in primary and secondary metabolism. These changes in the texture (softening of cell walls), colour (biosynthesis of pigments), taste and smell (accumulation of sugars and alteration of metabolomic profile) of the fruit usually make it more palatable for consumption (Goulao and Oliveira, 2008; Bianco et al., 2009; Palma et al., 2011; Tartaglia et al., 2021). Enzymes typically involved in the process of cell wall softening, such as polygalacturonase and endoglucanase (Jiang et al., 2019; Valenzuela-ri and Morales-quintana, 2019), were founded to accumulate in *A. unedo* fruits concomitantly with the actual textural change this fruit, which attains a soft texture only when fully ripe. Additionally, the marked increased abundance of chalcone synthase (CHS), the key enzyme in flavonoid biosynthesis through the phenylalanine metabolic pathway, in the fully ripe fruit indicates flavonoid accumulation in it. It is known from the literature that CHS gene expression is significantly correlated with pigment accumulation during fruit ripening and is influenced by many environmental factors, given the dramatic turning of *A. unedo* fruit from green to deep red in all its parts, the high green/red and veraisoned/red ratio of this protein is not surprising (Wang et al., 2010; España et al., 2014). As regards carbohydrate metabolism, an increased abundance of proteins such as sucrose-6-phosphate synthase and glucose-6-phosphate isomerase is observed during the *A. unedo* ripening; the accumulation of simple sugars such as sucrose, glucose, and fructose in the ripe fruit is essential for the achievement of the fruit’s organoleptic characteristics at the stage of consumption, both in terms of flavour and aroma. In fact, these carbohydrates act as precursors for several aroma compounds, as well as being a necessary source of energy for the ripening process (Li et al., 2019; Bhuiyan et al., 2020). As known from literature, generally the content of free amino acids in fruits increases during the ripening stages due to the high protein turnover triggered by the ongoing physiological process. Amino acid concentrations were closely related to anthocyanins and flavonols. However, amino acid content is influenced by the ripening stage and climatic conditions, which could be responsible for the different results obtained observing the same ripening stage of *A. unedo* fruits sampled in two different geographical areas (Campania/Sicily) (Sorrequieta et al., 2010; Ruiz-Rodríguez et al., 2017). The comparison of the fruit samples derived from the two sampling sites showed that from the point of view of protein abundance, the differences were due more to the ripening process than to the fruit harvesting site; on the other hand, the situation becomes more interesting from a metabolomic point of view. In fact, environmental conditions such as temperature and irradiance reshape not only the accumulation of sugars and amino acids but also the metabolome of the fruit, as the plant will adapt the phenotype to the environment (Reshef et al., 2017; Saini et al., 2019). In plants, lower amounts of flavonoids and polyphenols were often associated with climates with higher temperatures and reduced water availability, which cause the generic reduction in the concentration of secondary metabolites. Altitudinal differences also induce a difference in polyphenol content (Davies et al., 2010; Tomassini et al., 2016; Reshef et al., 2017; Saini et al., 2019). From the metabolomic profile analysis of the *A. unedo* fruits and leaves, the pedogeographic imprinting that shaped the expression of metabolites on landscape characteristics became evident. This variability in metabolic expression among individuals belonging to the same ecotype is attributable to environmental factors and agronomic conditions of the geographical area of origin, highlighting how a good characterization of niche plant species cannot transcend the contingent characterization of the environment.

## Data availability statement

The proteomic mass spectrometry data have been deposited with the ProteomeXchange Consortium via the partner repository PRIDE with the dataset identifier PXD041886.

## Author contributions

All authors make substantial contributions to conception and design of the manuscript. MT, RSci, CG and RSch conceptualized and designed the study. AG and APr collected the samples; MT, AA, AI, GP conducted the samples analyses. DZ, PS performed the statistical data analysis. All authors jointly interpreted data. MT wrote the first draft of the manuscript. All authors contributed to manuscript critical evaluation, revision, read, and approved the submitted version.
